# Pneumonia

**Published:** 1909-03

**Authors:** James B. Baird

**Affiliations:** Atlanta, Ga.; Professor Principles and Practice of Medicine, Atlanta College of Physicians and Surgeons


### Journal-Record of Medicine

Successor to Atlanta Medical and Surgical Journal, Established 1855, and Southern Medical Record, Established 1870.

**OWNED BY THE ATLANTA MEDICAL JOURNAL CO.**

**Published Monthly.**

*Official Organ Fulton County Medical Society, State Examining Board, Presbyterian Hospital, Etc.*

EDGAR G. BALLENGER, M. D., Editor.
BERNARD WOLFF, M. D., Supervising Editor.
A. W. STIRLING, M. D., C. M., D. P. H., J. S. HURT, B. Ph., M, D,
GEO. M. NILES, M. D., Associate Editors. E. W. ALLEN, Business Manager.

COLLABORATORS

DR. W. F. WESTMORLAND, General Surgery.
F. W. McRAE, M. D., Abdominal Surgery.
H. F. HARRIS, M. D., Pathology and Bacteriology.
E. B. BLOCK, M. D., Diseases of the Nervous System.
MICHEL HOKE, M. D., Orthopedic Surgery.
CYRUS W. STRICKLER, M. D., Legal Medicine and Medical Legislation.
E. C. DAVIS, A. B., M. D., Obstetrics.
E. G. JONES, A. B., M. D., Gynecology.
R. T. DORSEY, Jr., B. S. M. D., Medicine.
L. M. GAINES, A. B., M. D., Internal Medicine.
J. N. LeCONTE, M. D., Diseases of the Stomach and Intestines
L. B. CLARKE, M. D., Pediatrics.
EDGAR PAULIN, M. D., Opsonic Medicine.
R. R. DALY, M. D., Medical Society.
A. W. STIRLING, M. D., Etc., Diseases of the Eye, Ear, Nose and Throat.
BERNARD WOLFF, M. D., Diseases of the Skin.
E. G. BALLENGER, M. D., Diseases of the Genito Urinary Organs.

VOL. XI. MARCH, 1909. No. 12

### PNEUMONIA.*

BY JAMES B. BAIRD, M. D., ATLANTA.

PROFESSOR PRINCIPLES AND PRACTICE OF MEDICINE, ATLANTA COLLEGE OF PHYSICIANS AND SURGEONS.

Acute lobar pneumonia is an acute infectious disease. An infectious disease is one caused by the entrance and propagation in the living body of pathogenic micro-organisms. In this particular disease, the infectious agent is the pneumococcus, known also as the diplococcus of Fraenkel and as the micrococcus or bacillus lanceolatus.

Frequently associated with the pneumococcus, are the streptococcus, the staphyloccus an dthe baccilus of influenza, of diphtheria and of typhoid fever. The pneumococcus is often found in

*Read before the Fulton County Medical Society.

connection with lobular, catarrhal or broncho-pneumonia—though this type of the disease is usually due to a mixed infection, in which the bacillus of Friedlander holds a more or less important place.

Some bacteria produce more than one toxic agent, but the pneumococcus appears to be responsible for only one toxin, and the resulting toxaemia plays an important role in the clinical history of pneumonia.

It is confidently alleged by competent observers that the usual local expression of this infection—an exudative inflammation of the lung tissue—is not an essential part of the morbid process, but that a general pneumococcic sepsis may exist and run its course without the development of the ordinary local lesion in the lung. It is a well established fact that pneumonia is an essential fever, and that the inflammatory lesion is merely a local expression of a general condition.

Many years ago, that master medical mind—that great prophyetic sage, the late Austin Flint, wrote:

"Acute lobar pneumonitis, in the nosological systems of the present as of the past time, is placed among the local diseases, and in regard to certain questions it has been regarded as the type of a purely inflammatory affection. This view of its pathological character is now held to be erroneous. The pulmonary affection is doubtless inflammatory; but it is the local manifestation or the anatomical characteristic of an infectious febrile disease, sustaining to the latter a relation analogous to that which the affection of the solitary and agminated intestinal follicles sustains to typhoid fever." He continues: "If this doctrine be true, the proper place for the disease in the nosology is among the essential fevers. There are sufficient grounds for regarding the disease as an essential fever, and we may define it as a fever characterized anatomically by an abundant exudative deposit in the air vessicles of a single lobe or of two and sometimes three lobes of the lungs, with, in general, circumscribed bronchitis and dry pleurisy. It is a fever which rapidly reaches its maximum intensity, and has a short career the duration averaging about eleven days. It proves fatal chiefly in consequence of associated diseases, complications, or accidents, and, in the mode of dying asthenia usually predominates. It depends on a cause or on causes specific in character, the nature of which is not at present established.

It may be favorably modified, its duration abridged and the danger to life diminished by treatment addressed, not to the pulmonary affection, but to the fever."

Pneumonia is a frequent complication of other diseases, especially of typhoid fever, erysipelas, cerebro-spinal meningitis, dysentery and tuberculosis, and among the most common and the most important complications of pneumonia are meningitis, jaundice, acute arthritis and nephritis. It attacks all ages, but by preference young adults; men oftener than women; the vigorous and robust as well as the debilitated subject. It prevails in all countries; upon the mountain top and at the sea level. Its favorite season, in this section, is the early spring. One attack does not afford immunity from subsequent attacks, indeed, exactly the reverse is true. The susceptibility is increased after the first attack, and more than twenty distinct attacks in one individual are recorded.

Statistical data from many of the most populous cities of this country prove that it is now the most fatal of all infectious diseases, having within the last decade exchanged places with tuberculosis, which formely stood at the head of mortality lists.

The period of incubation is short, very short, probably not more than a few hours. The invasion is abrupt. The initial chill, which usually marks the advent of an attack, followed by high fever, a short, frequent, dry cough, rapid respiration, redness of one or both cheeks and sharp pain in the region of the nipple on the affected side direct attention to the chest, and these symptoms alone afford strong presumptive evidence of the existence of this disease. Soon, the occurrence, in most cases, of the crepitant rale with tenacious, rusty sputum—which are pathognomonic—and, later, dullness or flatness on percussion, with bronchial respiration and broncophony, establish the diagnosis.

In the majority of cases, the local morbid process commences in the lower lobe of the right lung. The lower lobe of the left lung comes next in order of frequency. Exceptionally, an upper lobe is primarily attacked. It is thought that unusual severity generally attends an attack beginning in an upper lobe. Independent involvement of the middle lobe of the right lung seldom occurs. When a second lobe is invaded it is not a mere extension of inflammation by contiguity, but it means the establishment of a new focus of infection. This advance may be indicated by increase of fever, but the chill pertaining to the original invasion is not repeated. Bronchitis in the affected lobe or lobes is always present, so, also is circumscribed, dry pleuritis. Except, rarely, when the superficial portion of the lobe is not involved in the inflammatory process, which begins at one or at several distinct points and extends from lobule to lobule until, in most cases, the entire lobe is included. Occasionally, however, conclusive evidence of the precise nature of an attack, which is afforded by physical signs, is delayed for several days after the development of the general symptoms. This is true of the, so-called, central pneumonia in which the morbid changes begin in the deeper portion of the lobe and only gradually approach the circumference. The so-called creeping pneumonia may manifest the same peculiarity and indeed, an attack sometimes appears to begin as an ordinary influenza, the physical signs of pneumonia not being appreciable for a week or more. Abundant herpes labialis is a very common symptom. Leucocytosis, more or less marked, is present from the beginning to the termination of the fever. The chlorides in the urine are greatly diminished or are wanting during the same period. Active, even violent, delirium may suddenly develop, and the attendant should be warned of this possibility and be prepared for the emergency. Most cases end abruptly by crisis, from the third to the twelfth day. If the attack should be prolonged beyond the twelfth day it indicates a probability of ending by lysis.

The essential and the dangerous element in pneumonia—as pneumonic fever—seems to be a specific toxaemia, and as there are no available means of correcting this condition—if we are without effective antidotes or antitoxins, it is possible at least, by timely resort to well-known therapeutic agents to promote tolerance, within the body, of this elusive poison, and, in the existing state of knowledge, it is only in this direction that we may look for success. Therefore, in the treatment of pneumonia, we may gracefully accept the fact that there is no certain prophylactic, and no specific medication. But this assertion is not, by any means equivalent to an admission that judicious management is not conducive to favorable results, or that all treatment is useless. Some of the prominent symptoms should be met, and familiarity with the natural history of the disease furnishes unmistakable clinical indications. The cough and pain usually require anodyne remedies. Insomnia may call for hypnotics. Fever deserves special attention, and the tendency toward asthenia affords ample ground for the employment of sustaining measures. Blood-letting, once thought so appropriate in this disease, is seldom indicated. Only very infrequently will distressing dyspnoea, associated with a full, bounding pulse, early in the attack, justify its use. The benefits formerly attributed to it may generally be secured by less objectionable means, and the loss of the precious fluid, indispensable to physical welfare, may thus be avoided.

It would be remarkable indeed—a veritable clinical anomaly—if sustained high temperature in this disease is, as has been alleged, not injurious but rather serviceable to the patient. Yet, strange as it may seem, this view has not only been advanced, but has been seriously defended, and to a limited extent has been accepted as true. If the temperature should remain at or about 103 F., it is, in my judgment, important to save the nervous system from the exhausting effect of this heat, and antipyretics should be employed for this purpose. Partial sponging with tepid water and the ice cap to the head are the means to be preferred, but if they fail, and, unhappily, they often do, drug antipyretics should not be withheld. The medical profession may have sins of omission as well as sins of commission justly laid at its door. Denying this relief, when it is clearly indicated, belongs to this category. I consider phenacetine, in suitable doses the best of this class. Properly employed, when not distinctly contra-indicated, by existing feebleness of the pulse, it is certainly harmless and positively beneficial. It does not act as a depressant when it subdues fever, allays distress and secures rest. If these palpable advantages follow its use, the effect contributes directly to support, and the drug would therefore seem to deserve credit for the conservation, not for the dissipation of strength and such, in fact, appears to be the case.

Thorough elimination deserves special attention. The presence of a potent poison in the blood is an important feature of an attack. It is assumed that the ordinary channels may be profitably employed for the removal of this toxin. Patients should be encouraged to drink water freely, and cathartics, as required, should be administered. Standing pre-eminent among all the drugs which are classed as eliminants—far above all others—is calomel. With its undoubted power to arouse the excretory functions, it holds an important place in the approved therapeutics of this disease.

Death comes to mankind either from failure of the circulation or of the respiration—from asthenia or from apnoea. It is rarely the case that respiratory failure determines the issue in pneumonia. The dreaded tendency is toward asthenia, and the recognition of this undisputed fact, furnishes the key to proper management. To be fore-warned is to be fore-armed, and with this knowledge, we are derelict if we defer the use of measures designed to avert this recognized tendency. A liberal, liquid diet should be provided and the early use of both tonics and stimulants—strychnine with whiskey or wine, in doses best tolerated—should always be advised. The latter to be omitted only in the event of an individual peculiarity adverse to their use. It is a grave mistake to wait for signs of failure before resorting to these measures. It is an unfortunate error of judgment to postpone acting until the evidence of exhaustion is apparent. We know, or ought to know that this danger is to be encountered, and it is our simple duty to forestall its development if we can.

In decided failure of the circulation as shown by increased frequency and diminished force of the pulse, digitalis may, with benefit, supplement other means. Its use should not be declined because of alleged inefficiency in febrile conditions. Progressive prostration may be met by increasing alcoholic stimulation regardless of the dose until the desired effect is obtained, remembering that in this disease, as in other infectious diseases, extra-ordinary tolerance of alcohol is sometimes manifested. Aromatic spirit of ammonia is a valuable adjunct on special occasions and strychnine, at this juncture, should be given hypodermatically rather than by the mouth. In approaching collapse, the effect of a solution of camphor in ether, hypodermatically, by its stimulating influence upon the nervous system, is sometimes truly remarkable.

Excepting sinapisms to the chest for pleuritic pain, I have long since discarded external applications. Blisters are only useful in the event of delayed resolution, when repeated small blisters over the affected part undoubtedly stimulate the tardy function of absorption. Padded and oil-silk jackets, poultices, plasters and the like are uncomfortable, inconvenient and of no value. This estimate applies alike to fomentations, to flaxseed meal and to a highly lauded—a so-called elegant compound of glycerine and white clay. By indorsement of this latest specific, an unsuspecting and innocent profession may largely augment the dividends of the enterprising manufacturers, and we may rest assured that our confiding patients are not the real beneficiaries.

Fresh air, and a plenty of it, is indispensable, but ordinary ventilation of the apartment occupied by the patient is sufficient; any excess is unnecessary if not injurious.

If the so-called open air treatment—out-of-door sleeping—is ever, under any circumstances, or in the treatment of any disease, advantageous, the conditions regarded as favoring its adoption can scarcely be compared with the conditions which obtain in acute pneumonia. Even in those pathological states in which the leading advocates of this out-door life claim most, they do not advise that any patient shall be suddenly thrust out of doors. The best exponents of the plan—but not their untutored imitators—urge the importance of gradually accustoming the patient to untried exposure, which wise precaution is inapplicable of course, in any acute illness of short duration.

Extreme measures as a rule are not to be commended. Conservatism and moderation represent a principle in therapeutics which it is highly desirable to recognize and to regard. If enough is enough, then it must logically follow that too much is too much. The "golden mean" is a proverbial phrase which possesses practical significance. But alas, this new method, this extreme method, this irrational method, this open air method presents spectacular features calculated to attract attention, to make talk and to impress the laity. It measures fully up to the dimensions of a fad and meets the requirements of an amazing and ever increasing demand for novelty.

If we will only exercise mental qualities with which we are all endowed in some degree, we may, at times, discover that we can do better than to blindly follow mistaken enthusiasts who assume the prerogatives of leadership. Nevertheless, when sheep begin to jump, the whole flock will heedlessly go through the gap with utter disregard of consequences.

Veratrum viride, once much used, is a remedy of genuine worth. It should be employed with caution, and it requires close watching. Extravagant claims have been made, of late, in favor of carbonate of creosote. Given in five-grain doses gradually increased to fifteen grains every two hours from the very inception of the attack, it is said to reduce the temperature, to quiet the circulation and to bring about a favorable and early termination.

No serum treatment, so far tried, has yielded satisfactory results. It is to be hoped and expected that the future will show material advances along this line.

Remedies designed to promote expectoration are not indicated. Only an inconsequential part of the exudate—sometimes none at all—is thus thrown off. In favorable cases, the deposit in the air cells is more or less promptly absorbed by the forces of nature, leaving the lung in tact. It may be removed in this way with great rapidity and without cough or expectoration.

As the sputum contains the specific organisms of the disease it is probable that due attention has not heretofore been paid to its complete disinfection.

The inhalation of oxygen gas and the hypodermic use of atropine appear to be of service in certain stages of the small pro-portion of cases characterized by the occurrence of cyanosis or by evident respiratory insufficiency.

Relapses rarely occur, and while pneumonia does not tend to eventuate in other diseases, it is a mistake to allow the convalescent to leave the bed or to resume the usual diet too soon.

